# Prospects of animal models and their application in studies on adaptive immunity to SARS-CoV-2

**DOI:** 10.3389/fimmu.2022.993754

**Published:** 2022-09-16

**Authors:** Xiaohui Wei, Na Rong, Jiangning Liu

**Affiliations:** National Health Commission Key Laboratory of Human Disease Comparative Medicine, Beijing Key Laboratory for Animal Models of Emerging and Remerging Infectious Diseases, Institute of Laboratory Animal Science, Chinese Academy of Medical Sciences and Comparative Medicine Center, Peking Union Medical College, Beijing, China

**Keywords:** SARS-CoV-2, animal model, adaptive immunity, cross-immune response, tissue-resident immune response, immune memory

## Abstract

The adaptive immune response induced by SARS-CoV-2 plays a key role in the antiviral process and can protect the body from the threat of infection for a certain period of time. However, owing to the limitations of clinical studies, the antiviral mechanisms, protective thresholds, and persistence of the immune memory of adaptive immune responses remain unclear. This review summarizes existing research models for SARS-CoV-2 and elaborates on the advantages of animal models in simulating the clinical symptoms of COVID-19 in humans. In addition, we systematically summarize the research progress on the SARS-CoV-2 adaptive immune response and the remaining key issues, as well as the application and prospects of animal models in this field. This paper provides direction for in-depth analysis of the anti-SARS-CoV-2 mechanism of the adaptive immune response and lays the foundation for the development and application of vaccines and drugs.

## Introduction

Severe acute respiratory syndrome coronavirus 2 (SARS-CoV-2) has been circulating since the end of 2019 and has infected more than five hundred million people and killed more than six million people (https://coronavirus.jhu.edu/map.html), which has had an impact on global public security and the global economy (1, 2). Although the development of vaccines and drugs has hindered the spread and pathogenic process of the virus, the emergence of a tremendous number of mutant strains (more than eight million), especially the delta and omicron strains, has reduced the protection afforded by existing vaccines and drugs, resulting in breakthrough infection (3–6). In response to the rapid spread of SARS-CoV-2, some countries, such as Ireland, Denmark, Sweden, the United Kingdom, and Iceland, hope to prevent infection and spread by raising the threshold of “herd immunity”, gradually regarding SARS-CoV-2 as similar to influenza and even implementing open-ended nonquarantine precautions. However, it cannot be ignored that we still know very little about SARS-CoV-2. Unlike severe acute respiratory syndrome coronavirus (SARS-CoV) and Middle East respiratory syndrome coronavirus (MERS-CoV) which were rapidly contained (7, 8), it remains to be investigated whether SARS-CoV-2 can be treated as influenza-like for prevention. Moreover, whether the emergence of new variants will increase the harm to public health safety remains worth exploring. Furthermore, how protective the existing vaccines and drugs are against the new variants and the durability of vaccine protection remain to be studied.

SARS-CoV-2 infection can initiate innate and adaptive immune responses. However, early studies have shown that patients with coronavirus disease 2019 (COVID-19) have extensive innate immune dysfunction (9–11), regarding type I and type III IFN responses, which are impaired and delayed, further increasing the clinical risk of patients (12, 13). At present, there is no evidence that the innate immune response can directly and completely control the early infection by SARS-CoV-2. The initiation of an adaptive immune response is critical for controlling SARS-CoV-2 infection and protecting the body from reinfection over time. Despite the uneven data cohort, overall, virus-specific antibody titers in people recovering from SARS-CoV-2 infection can be maintained for at least 4-6 months (14–16), and specific cellular immune responses last longer (17–19). The protective effect of SARS-CoV-2 primary infection on reinfection has received special attention. Follow-up studies on clinical patients have found that primary infection has a certain protective effect on the secondary infection (20).

The animal model is a very useful tool for clinical research on COVID-19. Various animal models of SARS-CoV-2 infection, including nonhuman primates (NHPs), rodents, etc. (21–30), have been developed and used to simulate the clinical pathology of the disease observed in humans and to test the preclinical effects of vaccines and drugs (31–35). Although animal models may not be able to recapitulate all the characteristics of human infection (36), they have more human-like characteristics than *in vitro* cell models and organoid models and can be used to indicate the pathogenesis of virus pathogenesis and immune protection mechanisms and can be used in other related research. In this study, we elaborated the research progress on SARS-CoV-2 *in vitro* and *in vivo* research models and adaptive immunology. In addition, we further summarized the application, advantages, and prospects of existing animal models in SARS-CoV-2 adaptive immunology research.

## Classification and application of SARS-CoV-2 infection models

**Figure 1:**
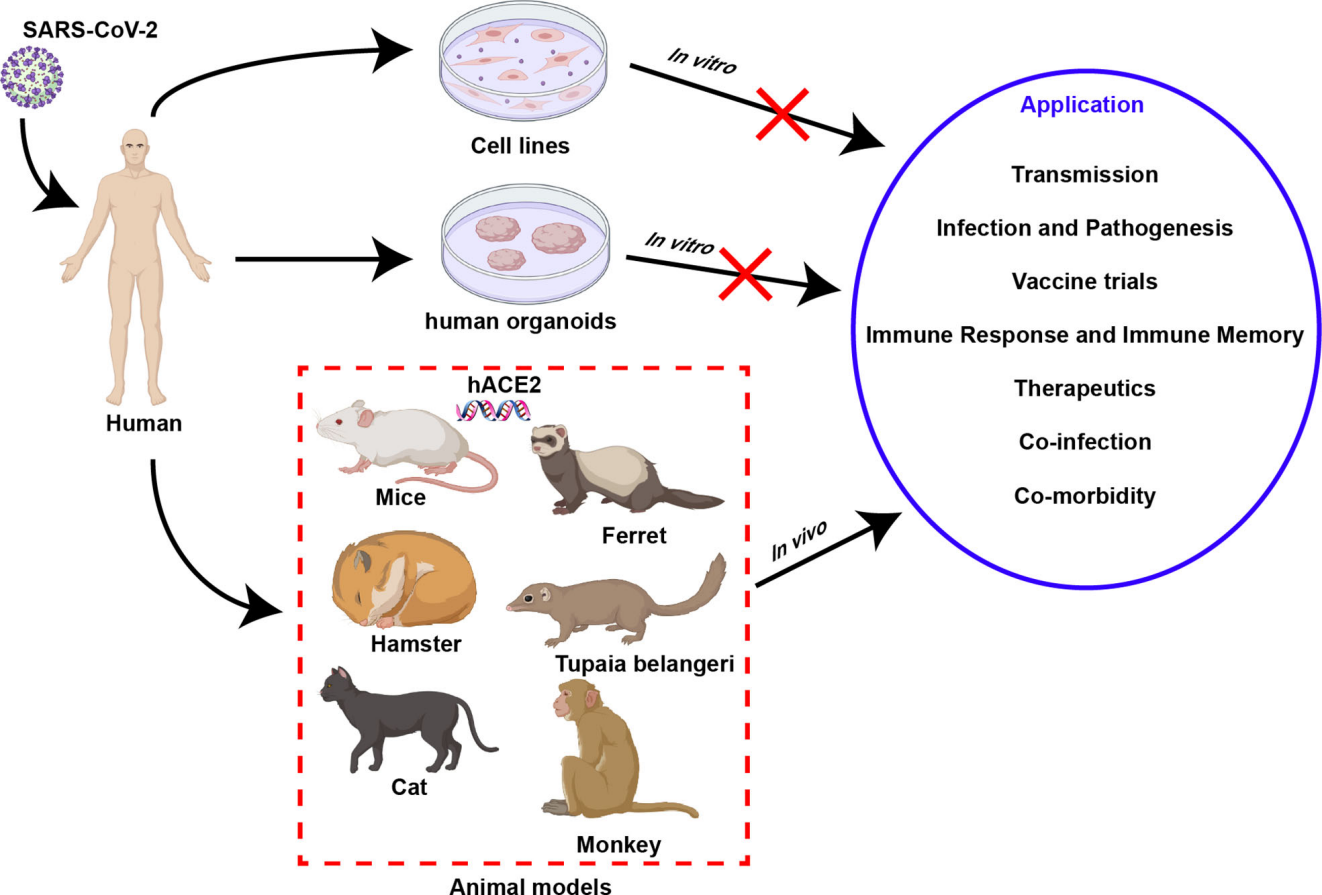
Models of SARS-CoV-2 infection. Three kinds of model can be used to study SARS-CoV-2, including *in vitro* model cell line, organoid, and *in vivo* animal models. In contrast with *in vitro* models, animal models can simulate virus-host interactions observed in human clinical research and have unique advantages in their application.

An in-depth understanding of SARS-CoV-2-related immunology is inseparable from the establishment and use of infection models. To date, three types of models have been established and applied in SARS-CoV-2-related research, including cells and organoids as well as animal models. They have different application scopes and advantages in related research **(** **Figure 1** **)**, which are elaborated on in this section.

### Cell lines

As a classic method to study viruses *in vitro*, cell lines are crucial for understanding the biology, growth kinetics, and tropism of SARS-CoV-2 infection. Compared with primary human bronchial epithelial cells, which can better reflect the characteristics of infection (37), passaged cell lines have a series of advantages such as infinite proliferation, high stability, low cost, and simple operation, and can be applied to virus infection and replication research. Human angiotensin-converting enzyme 2 (ACE2) has been identified as a key receptor for SARS-CoV-2 to bind and invade cells (38), so a variety of cell lines expressing ACE2 receptors have become effective tools for the study of SARS-CoV-2 **(** **Figure 1** **)**, such as Vero E6 cells (39), HEK-293T cells (39), Huh7 cells (40), Caco-II cells (41), and Calu3 cells (40). Especially in Vero E6 cells, the lack of type I interferon (IFN-I) and the high expression of the receptor ACE2 make it a cell line widely used to obtain high titers of virus particles of SARS-CoV-2 (42, 43). The cytopathic effects produced in virus-infected cell lines can be monitored for preclinical *in vitro* drug screening. However, the reliability of cell-based experimental results remains to be further investigated. For example, the antimalarial compounds chloroquine and hydroxychloroquine (HCQ) are effective in inhibiting the virus in cell lines, but less effective in primary cells and animal models (44–47). Therefore, although cell lines are convenient for studying SARS-CoV-2 *in vitro*, the obtained results still need to be further validated in primary cells and animal models. More importantly, deficiency immune-mediated or multicellular interactions are key factors limiting the application of cellular infection models.

### Human organoids

Compared with cell lines with good homogeneity, human organoids are more complex 3D structures composed of a variety of cells, which can better simulate the physiological state of normal human organs, so they are more suitable for the study of SARS-CoV-2 infection and tropism and drug screening *in vitro* (48). To date, a large number of human organoids have been developed and used in SARS-CoV-2-related research **(** **Figure 1** **)**, including lung organoids (49), bronchial organoids (50), kidney organoids (51), liver ductal organoids (52), intestinal organoids (53) and blood vessel organoids (54). Human organoids may allow observation of viral cell tropism, while exhibiting the accumulation of inflammatory cells and antiviral factors, reproducing the pathological symptoms after SARS-CoV-2 infection, which is crucial for understanding the pathogenesis of COVID-19 (55). Inevitably, the limitations of organoids prevent a deep understanding of severe COVID-19, possibly due to their lack of modulation of relevant immune components, including macrophages and natural killer cells. Furthermore, similar to the cell model, although neither can reproduce the pathogenic process of viral infection seen *in vivo* and the systemic antiviral infection process of the human body, they are good models for the convenient study of SARS-CoV-2 *in vitro*.

### Animal models

Compared with *in vitro* models, animal models for SARS-CoV-2 are more complex, but they can yield more reliable clinical data on virus-host interactions **(** **Figure 1** **)**. In detail, research on transmission routes, investigation of infection and pathogenic mechanisms, clinical evaluation of vaccines and drugs, elucidation of the host immune response and immune memory, the study of coinfection, and evaluation of complications, etc., requires the use of animal models **(** **Figure 1** **)** (56). Therefore, the rational development of animal models is crucial for gaining an in-depth understanding of SARS-CoV-2. To date, animal infection models that have been developed and are suitable for SARS-CoV-2-related research fall into five categories: small rodents, ferrets, cats, *Tupaia belangeris*, and NHPs.

Small rodents, including hamsters and genetically engineered mice, have many advantages such as easy operation, fast reproduction, and low cost, so they are widely used in research on the efficacy of antiviral drugs, vaccines, and immunotherapy. In particular, hamsters can be naturally infected by SARS-CoV-2 and show typical clinical symptoms, such as weight loss and multiple tissue and organ damage (57–59). Based on these characteristics, the hamster model has been applied to a series of studies, including transmission route (58), variant strain pathogenicity (60), and antibody therapy (61). Due to the limited specificity of the ACE2 receptor, wild-type mice are less sensitive to most variants of SARS-CoV-2, and only to the beta variant (62). Sensitization of mice by adenovirus expressing human ACE2 (Ad5-hACE2) is a fast and efficient method for susceptibility modeling and has been widely applied (63–66). Furthermore, genetically engineering mice to express the human ACE2 (hACE2) receptor is an effective way to increase their susceptibility to other variants of SARS-CoV-2. However, different genetically engineered mice have different clinical manifestations of disease due to differences in the modification and expression of the hACE2 receptors. For example, humanized mice expressing hACE2 under the epithelial cell-specific cytokeratin-18 (Krt 18) promoter (67), a universal chicken beta-actin (β-actin) promoter (68), or the human hepatocyte nuclear factor-3/forkhead homolog 4 (HFH4) promoter (25) are highly susceptible to SARS-CoV-2, develop severe pathological damage resembling COVID-19 symptoms in human lungs, and die rapidly. However, mouse models with only ACE2 replaced show only mild pathology and are confined to the lungs and intestine (24). Other nonprimate model animals, including cats (69, 70), ferrets (71), and *Tupaia belangeris* (72, 73), are susceptible to SARS-CoV-2 but do not produce severe clinical symptoms, so they may be potential intermediate hosts and ideal models for studying transmission in the absence of severe clinical symptoms.

The gold standard animal model that is closest to human genetics, anatomy, and immunology and is suitable for SARS-CoV-2 is the NHP model, mainly including African green monkeys (74), marmosets (23, 26, 75), baboons (75), cynomolgus macaques (23, 26) and rhesus macaques (26, 29, 30). They play a very important role in the study of the pathogenesis of COVID-19 and the development of vaccines and antiviral drugs. At present, it has been confirmed that the immune response of the NHP model after SARS-CoV-2 infection recapitulates the key features of COVID-19 in humans (76, 77). Therefore, the NHP model is the best model for evaluating drugs (78), neutralizing antibodies (79), and multiple vaccines (adenovirus, DNA, mRNA, inactivated, and subunit vaccines) (31, 80–85).

## Animal models mimic the clinical features and pathogenesis of COVID-19

COVID-19 is a series of clinical syndromes caused by SARS-CoV-2 in humans, including asymptomatic infection, mild-to-moderate upper respiratory tract infection, pneumonia, acute respiratory distress syndrome (ARDS), hyperinflammatory disease, and long-term neurological and cognitive dysfunction (86). The pathogenesis of COVID-19 is complex and its severity and pathogenicity are closely related to various factors, such as age, complications, and genetic or acquired factors (87–90). Evidence suggests that SARS-CoV-2 is highly persistent and dangerous to those with weakened immune systems, especially elderly patients with cardiovascular disease, diabetes, hypertension, and some other serious chronic diseases (86, 88, 91–93). In particular, elderly individuals >65 years old showed similar lymphopenia, neutropenia, inflammation, and coagulation-related index elevation (90, 94–98). While most young and pediatric patients are asymptomatic or have mild symptoms, many of them experience persistent fatigue, anhedonia, muscle weakness, sleep problems, anxiety and even depression, difficulty concentrating, myalgia, and arthralgia, autonomic function obstacles, etc. (89, 99–101). Although the mechanism remains unclear, SARS-CoV-2 may cause irreversible damage to the brain, based on the evidence that SARS-CoV-2 can infect the central nervous system and brain cells (102–104).

**Figure 2:**
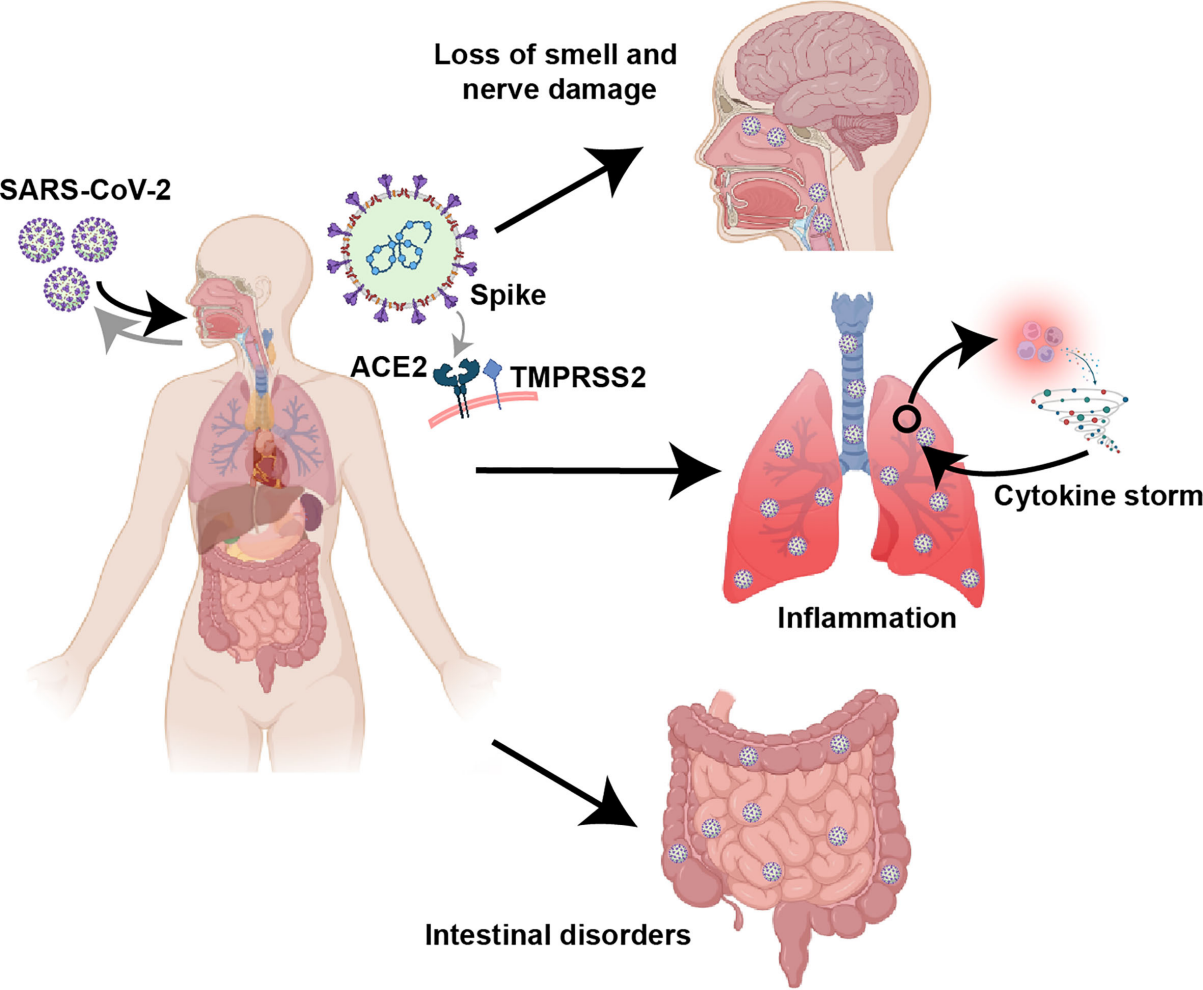
Clinical features of COVID-19. Based on the receptor (ACE2/TMPRSS2) binding specificity of the Spike protein, SARS-CoV-2 can infect multiple tissues and organs, especially the respiratory tract, lung and intestine. COVID-19 can cause significant lung inflammation, intestinal disturbances, and nerve damage, including loss of smell.

Viral tropism depends on the susceptibility and permissiveness of specific host cells. Some studies suggest that SARS-CoV-2 induces an inflammatory response in the lower respiratory tract leading to lung injury **(** **Figure 2** **)**, confirming that the lung is the main site of infection by SARS-CoV-2 (105, 106). Unlike SARS-CoV, SARS-CoV-2 can efficiently infect the upper respiratory tract (URT), such as nasopharyngeal and oropharyngeal tissues, increasing its infectivity and pathogenicity **(** **Figure 2** **)** (107–110). In addition, the gut is another target of SARS-CoV-2, and patients with COVID-19 often report suffering from gastrointestinal diseases **(** **Figure 2** **)** (111, 112). It is generally believed that the classical mechanism of SARS-CoV-2 infection targeting host cells occurs through the interaction between the viral surface structural protein Spike and the cell surface protein ACE2 (38). In addition, the function of the Spike protein requires the assistance of transmembrane serine protease 2 (TMPRSS2) (38, 113). Although it has been reported that other receptors or cofactors are also involved, such as the endosome/lysosomal cysteine proteases cathepsin B and L (CTSB, CTSL) (38), CD147 (114, 115), Neuropilin 1 (116, 117) and DPP4 (118, 119), whether these factors are necessary for SARS-CoV-2 invasion remains to be further confirmed. At least at the level of host protein expression, it has been determined that ACE2/TMPRSS2 are coexpressed in multiple tissues, including nasal epithelial cells (120), the alveolar epithelium (mainly type 2 alveolar cells), the bronchial branch epithelium (120, 121), the intestinal tract (122, 123) and the nervous system (124). This finding is also in line with the infection characteristics of SARS-CoV-2 and the clinical characteristics of patients.

Animal models can mimic the clinical symptoms of COVID-19 in humans and are good models for studying the pathogenesis caused by SARS-CoV-2. A study showed that hACE2-HB-01 transgenic mice exhibited significant weight loss, supported viral replication, and displayed lymphocyte and monocyte infiltration in the alveolar interstitium and macrophage accumulation in the alveolar space after infection with SARS-CoV-2 (24), which recapitulate clinical patient findings (125). Furthermore, many studies have shown that Krt18-hACE2, β-actin-hACE2, and HFH4-ACE2 transgenic mice die rapidly after infection with SARS-CoV-2. Their lungs have high viral loads and severe interstitial lesions caused by inflammatory immune cell infiltration, with marked thrombosis and anosmia and neuroinvasive symptoms, consistent with human COVID-19 (25, 68, 126–128).

In the hamster model, SARS-CoV-2 infection has been found to cause weight loss, ruffled fur, postural changes, and symptoms similar to those of human COVID-19, such as dyspnea, olfactory epithelial cell and neuron damage, severe lung pathology, and lymphopenia (59, 129, 130). Furthermore, older hamsters exhibit more pronounced weight loss than younger hamsters (131). Although virus titers are higher in young mice, rapid clearance of the virus can be observed (131). In addition, SARS-CoV-2 infection can occur in hamsters through direct contact, aerosol transmission, and oral transmission, so this model is often used to evaluate the airtightness of surgical masks (58, 132, 133). Moreover, hamsters can be a valuable tool to study the correlation between SARS-CoV-2 pathogenicity and age.

Ferrets are considered the best model for asymptomatic or mild diseases in humans and are frequently used in transmission studies. Multiple studies have shown that ferrets infected with SARS-CoV-2 experience rapid transmission before peak viral load and body temperature, while the virus replicates primarily in the upper respiratory tract of ferrets without causing severe disease, which is consistent with the characteristics of individuals during asymptomatic periods (21, 134, 135). Multiple additional studies showed that ferrets can mimic both direct and indirect transmission of COVID-19 in humans (71, 134, 135). A profile of inflammatory cytokines similar to that in humans, including the expression of genes encoding IL-6, IL-1β, CCL2, and CCL8, was observed in the airways of ferrets after infection (136). In addition, compared with juvenile and adult ferrets, aged ferrets have higher viral loads, longer shedding times, more severe inflammatory cell infiltration in the lungs, and more clinical symptoms (137). In another model animal, *Tupaia belangeri*, higher titers were also observed early after infection in young tree shrews but for a longer duration in elderly tree shrews (73). In addition, evidence of an inflammatory process in the lungs was found in all age groups, and infiltration was similar to that observed in humans and monkeys (72).

The NHP model is the closest model to humans. At present, NHP models applied to SARS-CoV-2 research mainly include African green monkeys (74), marmosets (23, 26, 75), baboons (75), cynomolgus macaques (23, 26), and rhesus macaques (26, 29, 30). Among them, rhesus monkeys showed clinical symptoms similar to COVID-19 patients after infection, including increased expression of inflammatory cytokines and chemokines, decreased blood oxygen levels, and decreased white blood cell and lymphocyte counts (26, 29, 30, 75, 77). Meanwhile, several studies have also shown that lower doses of SARS-CoV-2 infection in African green monkeys than those infected with rhesus monkeys can still cause significant viral replication and lung lesions, which indicates that SARS-CoV-2 also has pathogenic potential in African green monkeys (74, 138). In addition, although there are fewer studies on baboons, one study showed that baboons are sensitive to SARS-CoV-2 infection and develop extensive pathological changes after infection (75). For marmosets and cynomolgus monkeys, the clinical symptoms of SARS-CoV-2 infection were mild and manifested as mild pneumonia (23, 26, 75). Although different monkey species in the NHP model differed in susceptibility and pathogenicity to SARS-CoV-2, overall they did not develop the characteristic cytokine storm syndrome and respiratory distress or even death similar to severe COVID-19 patients (23, 29, 139). In summary, NHP models, especially rhesus monkeys, African green monkeys and baboons, play a very important role in the testing of vaccines and antiviral drugs and the study of virus mechanisms.

## The adaptive immune response and immune memory against SARS-CoV-2

**Figure 3:**
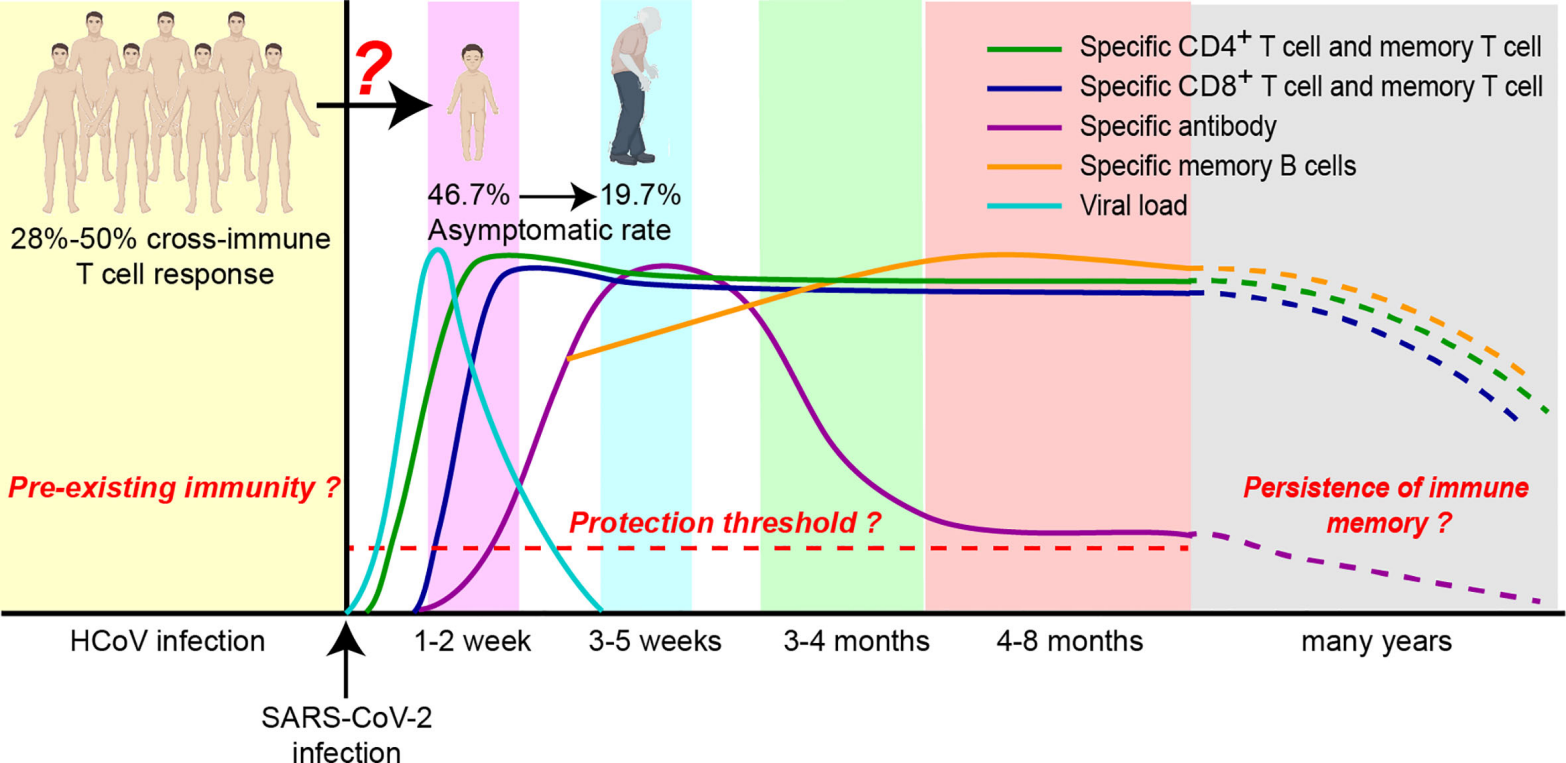
Persistence of adaptive immune responses to SARS-CoV-2. Following infection with SARS-CoV-2, an adaptive immune response is initiated. The viral load peaks at one week, CD4^+^ and CD8^+^ T cells peak at 1-2 weeks, and antibody levels peak at 3-5 weeks. The persistence and protective thresholds of immune memory in cellular and humoral immune responses are currently unknown. The association of preexisting immunity elicited by cross-reactive T-cell immune responses with asymptomatic infection remains to be further investigated.

Dysregulation of the innate immune response is closely associated with failure to control primary SARS-CoV-2 infection and a high risk of fatal COVID-19 (9–11). Although the adaptive immune response develops slower than the innate immune response, it responds to pathogens in an antigen-specific manner to generate protective immunity capable of protecting the host from reinfection with SARS-CoV-2 (140–142). This indicates that the adaptive immune response, including the action of B cells, CD4^+^ T cells, and CD8^+^ T cells, plays a key role in the antiviral process **(** **Figure 3** **)**. This subsection will elaborate on the adaptive immune response elicited by SARS-CoV-2 and the key role of animal models in facilitating related research.

### Humoral immune response against SARS-CoV-2

**Figure 4:**
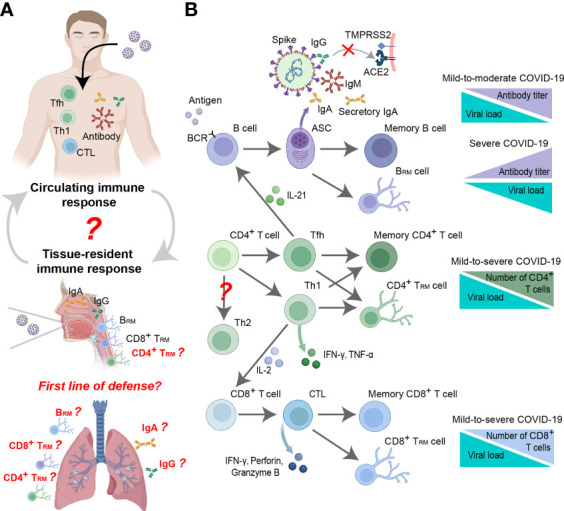
Antiviral mechanism of the adaptive immune response to SARS-CoV-2. **(A)** *In vivo* distribution of virus-specific adaptive immune responses, including circulating and tissue-resident adaptive immune responses. The relationship between circulating immune responses and tissue-resident immune responses remains to be further investigated. The role of lung tissue-resident immune responses in the fight against SARS-CoV-2 infection is worth investigating. **(B)** Mechanisms of adaptive immune responses against SARS-CoV-2 and their relationship to disease severity.

Neutralizing antibodies produced by adaptive humoral immune responses play a unique role in inhibiting virus invasion and killing target cells (143). Studies have shown that approximately 90% of the sera collected from COVID-19 patients on the 10th day after infection carry specific antibodies against the Spike and N proteins **(** **Figure 3** **)** (144–148), and specific antibodies against the Spike protein, especially the receptor-binding domain (RBD), can exert a significant neutralizing effect (61, 148, 149). Neutralizing antibody levels peak within the next 3-5 weeks and then decrease, reaching the lower threshold of detection within months **(** **Figure 3** **)** (14, 16, 150–152). Although neutralizing antibody titers drop rapidly, these antibodies still act immediately when the virus re-enters the host. Residual neutralizing antibodies on the mucosal surface can directly block the initial infection of host cells while circulating neutralizing antibodies can further prevent the occurrence of infection **(** **Figures 4A, B** **)**. One study showed that a neutralizing titer equivalent to 20% of the mean titer during the recovery period was sufficient to provide 50% protection (153). In addition, non-neutralizing virus-specific antibodies may contribute to immune control of infection by increasing clearance of free virus or by targeting infected cells for immune clearance (through antibody-dependent cytotoxicity and other mechanisms) (154–156), although this phenomenon is difficult to quantify (157, 158). The mechanism may occur through Fc receptor-related functions in serum. Previous studies have confirmed that neutralizing antibodies with Fc receptor-binding ability are more protective in mice, while Fc-dependent antibody effector activity in non-surviving humans has been found to be reduced (82, 154, 159). Moreover, non-neutralizing antibody Fc effector functions such as complement activation, cytotoxicity, and phagocytosis have also been shown to protect model animals (154, 156).

The specific antiviral effects exerted by neutralizing antibodies make them potential therapeutic drugs in the clinic. One study demonstrated that administration of SARS-CoV-2-specific monoclonal antibodies in the early stages of infection in patients with mild-to-moderate COVID-19 accelerated the virus clearance and reduced clinical risk **(** **Figure 4B** **)** (160, 161). It has also been found in animal models that human neutralizing antibodies can protect hamsters and mice, reduce clinical symptoms and even prevent infection (59, 61, 154, 162). In addition, the administration of neutralizing antibodies in NHP models can yield prophylactic and protective effects (79, 162, 163). However, the clinical manifestations of specific monoclonal antibodies administered to critically ill patients are quite different, and some studies have shown that increasing antibody levels can improve clinical outcomes (164, 165), while other studies have reported poorer clinical outcomes **(** **Figure 4B** **)** (166, 167). This discrepancy may be related to the antibody-dependent enhancement of COVID-19 (168), which is supported by *in vitro* and animal experiments (169–175). Interestingly, SARS-CoV-2 neutralizing antibody titers are independent of primary COVID-19 disease severity (145, 176, 177). Based on various animal models, neutralizing antibody titers (and total anti-Spike antibody titers) were further confirmed to be positively correlated with COVID-19 disease severity **(** **Figure 4B** **)** (149, 178). One possible reason is that high neutralizing antibody titers are associated with severe disease and underlying extrafollicular B-cell responses (179).

Neutralizing antibody is a double-edged sword, but whether they are necessary to control SARS-CoV-2 infection remains controversial. One study showed that COVID-19 patients with agammaglobulinemia and no circulating B cells still fully recovered from infection (180), and B-cell depletion therapy in COVID-19 cases have also shown some efficacy (181–183). Furthermore, some convalescent patients have not had detectable neutralizing antibodies against the virus throughout the disease course (18, 145, 184, 185), which supports the view that a humoral immune response may not be necessary to control SARS-CoV-2 infection.

Nonetheless, most of the current COVID-19 vaccine efforts are still focused on the induction of the production of neutralizing antibodies (186–188). The favorable therapeutic effect of neutralizing antibodies in clinical and animal models (59, 160, 162) and the increase in the number of B cells in convalescent patients (189) make the development of vaccines more promising. However, SARS-CoV-2 has a high level of genetic variability, producing mutant strains such as the alpha strain, beta strain, gamma strain, delta strain, zeta strain, and omicron strain, and the total number of mutants has exceeded 11 million (https://ngdc.cncb.ac.cn/ncov/release_genome). Among them, the number of omicron strains has reached nearly 4 million, and the number is growing continuously. Mutations focused on the Spike protein, especially the RBD, have received widespread attention, as they can allow immune evasion by reducing or even failure of the protection afforded by neutralizing antibodies (190–194). In addition, the high glycosylation level of Spike proteins promotes this effect (195–197). Notably, there are reports that partially neutralizing antibodies can cross-protect against multiple variants (198, 199), providing some promising reports for vaccine development. However, the absence of neutralizing antibodies does not mean that immunity is invalid, and the antiviral effects of neutralizing antibodies at different stages of the disease still need to be further confirmed by clinical data. Specifically, when the neutralizing antibodies produced during primary infection or vaccine immunization disappear, will the neutralizing antibodies secreted by immune memory B cells in a short period during reinfection provide timely effective protection? Furthermore, whether neutralizing antibodies are sufficient to provide complete protection in the absence of a cellular immune response remains to be supported by data. At present, such data are lacking in clinical practice, and it is also difficult to perform targeted research, so the corresponding clinical data must be obtained only *via* animal models with insufficient T-cell immune responses.

### Cellular immune response against SARS-CoV-2

As an important part of the adaptive immune response, the cellular immune response also plays an important role in countering SASR-CoV-2 infection. Neutralizing antibody titers are independent of the severity of primary COVID-19 (16, 145, 149, 176–178), while the magnitude of virus-specific T-cell responses is highly correlated with disease severity **(** **Figure 4B** **)** (18, 145, 200, 201). In addition, T-cell responses have been detected in almost all COVID-19 patients (18, 19, 145, 202), suggesting that T-cell responses may be the key to the control and resolution of primary SARS-CoV-2 infection.

Although the data are not completely consistent, some studies have confirmed that CD4^+^ T cells exhibit a more prominent response and detection rate to SARS-CoV-2 than CD8^+^ T cells (18, 19, 203) and are associated with the control of primary SARS-CoV-2 infection (145, 177), especially circulating T follicular helper cells (cTfhs) (145). This may be related to the function of cTfhs, which play a key role in the development of long-term humoral immunity through the germinal center response and are essential for the development of most neutralizing antibody responses as well as memory B cells and long-term humoral immunity **(** **Figure 4B** **)** (203–205). At present, many studies have shown a correlation between the intensity of the SARS-CoV-2-specific antibody response and the CD4^+^ T-cell response (19, 206). In addition, CD4^+^ T cells can also differentiate into other effector cells with more direct antipathogen activity, Th1 cells, which function mainly by secreting IFNγ **(** **Figure 4B** **)** (19, 145, 207, 208). CD4-cytotoxic T lymphocytes (CTLs) are a related cell type with direct cytotoxic activity, and although some studies have observed CD4-CTL transcriptional signatures (209), their function remains unclear. Notably, Th2 cells are extremely rare in patients with COVID-19 (18, 19, 145) and appear only in critically ill patients (210). Whether this is related to the aggravation of disease in critically ill patients remains to be explored.

SARS-CoV-2-specific CD4^+^ T-cell responses can be detected as early as 2-4 days after infection **(** **Figure 3** **)** (145, 177, 208) and can produce specific responses to almost all viral proteins, especially the Spike, M, and N proteins, during the recovery period, which are correlated with protein expression levels (19). This finding is very promising, as most of the current vaccine candidates focus on Spike (211). In addition, studies based on earlier animal models of SARS-CoV and MERS-CoV infection confirmed that in the absence of antibodies or CD8^+^ T cells, adoptive transfer of CD4^+^ T-cells has a protective effect (212). This finding suggests that the cellular immune response, especially the CD4^+^ T-cell immune response, may be the key to controlling SARS-CoV-2. In addition, in-depth studies of CD4+ T cell responses based on the NHP model also reconfirmed that SARS-CoV-2 was able to induce a robust germinal center CD4^+^ T follicular helper cell response (213).

CD8^+^ T cells can kill infected cells and are also critical for clearing viral infections. Studies have shown that CD8^+^ T cells in patients with COVID-19 are usually observed within 7 days of symptom onset, with level peaking at 14 days **(** **Figure 3** **)** (210, 214), and are more activated than CD4^+^ T cells (142, 215, 216). Furthermore, some studies have demonstrated that CTL reactivity is associated with improved clinical outcomes in COVID-19 patients **(** **Figure 4B** **)** (145, 203, 210, 214, 217). Similar to SARS-CoV-2-specific CD4^+^ T-cells, CD8^+^ T cells develop rapidly in the acute phase (145) and produce good antigen specificity against the Spike, N, M, and ORF3a proteins of SARS-CoV-2 (18, 19, 185, 202, 218). Moreover, several studies have reported that intensive care unit (ICU) patients have significantly fewer CD8^+^ T cells than CD4^+^ T cells, which appears to be associated with COVID-19-related disease severity and mortality (91, 219–222). Therefore, many vaccines are devoted to stimulating T cell responses and achieving good anti-SARS-CoV-2 infection effects in animal models, including mRNA vaccines (223–225), lentiviral vaccines (226), adenovirus vaccines (83, 227, 228), etc.

Although the T-cell response may be the key to controlling and resolving primary SARS-CoV-2 infection, it is worrisome that approximately 82.1% of SARS-CoV-2 patients develop lymphopenia (229), and the degree of reduction of circulating T-cell levels is as high as 80% (11, 230). This finding suggests that the cellular immune response is suppressed, affecting the clinical recovery process (91, 222, 231). The mechanism leading to lymphopenia in SARS-CoV-2 patients is still unclear and remains to be explored *via* animal model studies. Nonetheless, SARS-CoV-2 variant strains do not escape T-cell immunity as they escape humoral immunity (190–193) because the T-cell epitopes of SARS-CoV-2 are very extensive and involve almost all viral proteins (19, 232–235). In addition, several studies have also shown that T-cell immunity induced by the SARS-CoV-2 vaccine can cross-recognize multiple mutant strains (236, 237), which may be promising for vaccine design and development. Based on the above results, we are optimistic about the relevant research on T-cell epitope vaccines in animal models and determining whether they can achieve the immune protection effect across variant strains. In addition, the current direction should also advance research on antibody-independent T-cell immune protection and immune memory. From previous experience, the adoptive transfer of CD4^+^ T cells has a protective effect against SARS-CoV and MERS-CoV infection (212), but data on SARS-CoV-2 are very scarce. It is urgent to study the immune protection and immune memory of CD4^+^ T cells or CD8^+^ T cells alone or in combination using genetically engineered mouse models lacking humoral immune responses or other models based on short-term antibody clearance of humoral immune responses.

### Cross-immune response against SARS-CoV-2

Large-scale survey studies have shown that approximately 20-40% of SARS-CoV-2-infected people suffer from the asymptomatic disease (238–240), and asymptomatic disease is linked to age **(** **Figure 3** **)**. The incidence of asymptomatic manifestations was 19.7% in elderly individuals and 46.7% in children **(** **Figure 3** **)** (241). However, the mechanism of this discrepancy remains unclear but may be related to preexisting cross-reactive immune responses. SARS-CoV-2 is a member of the coronavirus family that also includes the human coronaviruses (HCoVs) HCoV-OC43, HCoV-HKU1, HCoV-229E, and HCoV-NL63, which cause the common cold, as well as SARS-CoV-1 and MERS-CoV. Studies on cross-reactive humoral immune responses confirm that cross-neutralizing antibodies to Spike proteins are extremely rare (148, 242–245). Surprisingly, cross-reactive memory T cells are frequently present, detectable in 28%–50% of the population **(** **Figure 3** **)** (19, 207, 208, 218, 246, 247), and the vast majority are cross-reactive CD4^+^ memory T cells (19). Their recognition epitopes are mostly located in common cold-causing coronaviruses. Although cross-reactive memory CD8^+^ T cells have been observed less frequently (19), they are still biologically relevant (184). The marked differences in cross-reactive CD4^+^ T-cell and CD8^+^ T-cell responses may be related to the restricted presentation pattern of major histocompatibility complex class I and II molecules (248, 249). Interestingly, some studies have shown that cross-reactive CD8^+^ T-cell responses are mainly localized in tissues but are rarely detected in the circulatory system and may represent the first line of defense in adaptive immune responses **(** **Figure 4A** **)** (250, 251). In a mouse model, studies have also confirmed the existence of cross-reactive T cells after SARS-CoV-2 infection, and mainly cross-reacted with SARS-CoV but not MERS-CoV (65).

Currently, the clinical importance of preexisting cross-reactive T-cell responses in the control of SARS-CoV-2 infection and the impact on herd immunity thresholds are controversial, as the protective mechanisms remain unclear (246, 252). Some studies, after controlling for age and other factors, have found that SARS-CoV-2 patients with a confirmed HCoV infection in the past 3 years had a significantly lower risk of being admitted to the ICU (253). In addition, individuals with strong HCoV-specific T cells may acquire excellent protective cellular immunity after exposure to SARS-CoV-2 (253). Other studies argue that HCoV-specific T cells generally have a low affinity for SARS-CoV-2 peptides (254, 255), making it difficult to play a role in controlling SARS-CoV-2 infection. Regardless of which perspective is correct, further research is urgently needed to map the cross-reactive memory T cells against SARS-CoV-2 induced by different coronaviruses based on animal models under strictly controlled conditions to evaluate their cross-protection efficiency. This research direction has important implications for the control of disease spread and the potential for herd immunity.

### Tissue-resident immune response against SARS-CoV-2

The circulating adaptive immune response plays a key role in the body’s defense against SARS-CoV-2 infection, but there are very limited reports on the functions of tissue-resident immune cells residing in nonlymphoid tissues including the lung and URT. Compared with circulating central memory cells and effector memory cells, tissue-resident memory cells respond faster, so the presence of tissue-resident memory cells may be critical for efficient target recognition and immune recall (256, 257). For SARS-CoV-2 infection, some studies have shown that circulating immune responses largely reflect local immune responses, and IgG and IgA antibodies against Spike in saliva have been found to correlate with antibodies in the blood **(** **Figure 4A** **)** (258). However, the neutralizing ability of secretory IgA produced by tissue-resident memory B (B_RM_) cells is stronger than that of circulating IgA, which may be related to its function and dimeric structure **(** **Figure 4A** **)** (259, 260). The respiratory tract is the main tissue invaded by SARS-CoV-2, so it is urgent to confirm the protective effect of B_RM_ cells in respiratory tissues against reinfection. Intranasal lentiviral vector-mediated antibody delivery reduces SARS-CoV-2 infection in aged and immunocompromised mice, further confirming that antibodies play a non-negligible role in mucosal immunity (261). Over the years, intranasal vaccination has been shown to induce airway IgA and reduce viral load in the early stages of viral infection (262). Therefore, several SARS-CoV-2 intranasal vaccines including lentiviral vaccine (226), adenovirus vaccine (83, 227, 228), and adjuvant-assisted subunit vaccines (263, 264) were developed. Based on animal models, it can elicit systemic and pulmonary antibody responses and significantly reduce pulmonary viral load and local lung inflammation (83, 226–228, 263).

Tissue-resident memory T (T_RM_) cells may also play a key role locally against SARS-CoV-2 viral infection. Studies have shown that SARS-CoV-2 infection can stimulate the production of T_RM_ cells in local tissues (nasal cavity and lung), and their quantity and degree of activation are associated with clinical protection (265, 266); these cells can last for at least 10 months or more **(** **Figure 4** **)** (267). Another study based on bronchoalveolar lavage samples from COVID-19 patients showed that there were more T cells in the lungs in moderate cases than in severe cases (201), which may be related to the degree of T-cell response impairment and the number of infiltrating cells (268). Studies based on cross-reactive T_RM_ cells have also reported that CD8^+^ T_RM_ cells may act as the first line of defense in the adaptive immune response against SARS-CoV-2 infection **(** **Figure 4A** **)** (250, 251). Other studies have presented the opposite view, arguing that T_RM_ cells in lung tissue are not sufficient to produce anti-infective protection against SARS-CoV-2 (269, 270). A study based on the NHP model demonstrated that vaccine-induced strong neutralizing antibody and CD8^+^ T-cell responses can protect the lungs from SARS-CoV-2 infection and viral replication early in infection (days 2-4) (271). The same results were also confirmed in mice (83, 226, 263). These findings suggest that local tissue-resident memory cells are beneficial in antiviral responses **(** **Figure 4A** **)**. However, the relationship between tissue-resident and circulating memory cells and their migration during SARS-CoV-2 infection remains unclear. Although some studies have shown that tissue-resident memory CD8^+^ T cells can shape local and systemic secondary T-cell responses (272, 273), more evidence related to SARS-CoV-2 is still needed.

However, studies on the actual protective effects of B_RM_ and T_RM_ cells are difficult to perform in the clinical setting because it is difficult to separate pulmonary B_RM_ and T_RM_ cells and the interference of many uncontrollable external factors cannot be excluded. Therefore, the animal model of SARS-CoV-2 infection has become a good model for studying tissue-resident immune responses, which can provide conditions for investigating the protective effect and immune memory of tissue (including nasal cavity and lung)-resident immune responses. Notably, tissue-resident immune responses and immune memory can be initiated only by natural infection and nasal vaccination, while other vaccination routes are difficult to efficiently initiate such responses. We are optimistic about research on the immunoprotective effect of the tissue-resident immune response in the initial stage of SARS-CoV-2 infection. This aspect is important because if the tissue-resident immune response is sufficient to protect the body from infection in the early stages of SARS-CoV-2 infection, the vaccine should be replaced and the nasal route is recommended. On the one hand, this route of vaccination is expected to prevent the introduction of pathogens and damage, and on the other hand, it can effectively hinder viral spread. However, evaluation of the effectiveness of the corresponding vaccine has higher requirements for the animal models of simulated infection, such as the need for sensitivity to aerosol or low-dose nasal infection, while animal models of high-dose infection and intratracheal infection cannot be used to evaluate nasal inoculation vaccines. Krt18-hACE2, β-actin-hACE2, or HFH4-ACE2 transgenic mice, golden hamsters, etc., are good animal models for studying tissue-resident immune responses.

### Immune memory and protection against reinfection

Immune memory is the source of protective immunity against SARS-CoV-2 reinfection, and it is one of the issues that has been widely studied. Therefore, in addition to measuring circulating antibody levels, evaluating specific memory B- and T-cell persistence may be critical for understanding and predicting the persistence of protection against SARS-CoV-2 infection.

Multiple studies have shown that the frequency of circulating memory B cells in COVID-19 patients continued to increase during the first few months, peaking at 6 months **(** **Figure 3** **)** (16, 274). Moreover, the generated memory B-cell pool for Spike proteins is predominantly IgG and more potent (16, 274). The measurement of T-cell immune memory at 6 months post-infection showed that the positive ratios of memory CD4^+^ T cells and memory CD8^+^ T cells were 90% and 70%, respectively, and the proportion of memory CD4^+^ T cells was nearly double that of memory CD8^+^ T cells (16, 150, 275). However, the half-lives of both may be 3-5 months. Through long-term follow-up monitoring of recovered patients, it has been found that the persistence of SARS-CoV-2-specific T-cell responses is better than that of humoral immunity (17). Virus-specific memory T cells can still be detected when the serum antibodies of patients with asymptomatic infection and mild disease are negative (18, 275). In fact, based on previous studies on SARS-CoV (218, 276, 277) and MERS-CoV (278), memory T cells specific for SARS-CoV-2 are expected to be maintained for many years (150). Although the impact of specific immune responses mediated by memory T cells on the pathogenesis of COVID-19 is unclear, based on the results of the assessment of cross-reactive T cells (19, 279), preexisting cross-immune memory may confer resistance to asymptomatic infected individuals. This information will help guide the design of broad-spectrum protective vaccines.

Indeed, whether the immune memory of patients recovering from COVID-19 can protect against reinfection with SARS-CoV-2 remains an open question. It is worrisome that some studies have reported secondary infections. In a comprehensive analysis of more than 133,000 cases, 54 were thought to be reinfections with SARS-CoV-2 at least 45 days after initial infection, and only four were infections with a different variant (280). Another survey study of more than 43,000 participants found that the reinfection rate in seropositive individuals was only 5%. In addition, by examining more than 500,000 people, it was found that patients after recovery from COVID-19 had 80.5%protection for up to 7 months, although protection was observed in only 47% of patients over 65 years of age (281). Although the infection rate is low, a seroepidemiological study also suggested that reinfection is possible 6 to 8 months after infection (282–284), especially since the rapid mutation of the virus may further increase the risk of reinfection (284, 285).

SARS-CoV-2 has been spreading continuously for nearly 3 years since it was first reported, and it has been regarded as influenza in many countries, with no preventative measures. Therefore, it may not be eliminated as quickly as SARS-CoV and MERS-CoV (7, 8). The maintenance time and protection threshold of vaccines and immune memory generated after infection need to be further studied. Although it is difficult to perform relevant experimental studies in clinical practice, because animal models simulate the main clinical symptoms of COVID-19 patients (36), they have become a good tool to study the SARS-CoV-2 immune response and immune memory. The NHP model, in particular, has unique advantages in clinical research (29). Studies have found that primary infection with SARS-CoV-2 in the NHP model protects against high-dose viral challenge for at least 28-35 days, and re-exposure drives memory immune responses that include high levels of binding neutralizing antibodies (29, 30). Furthermore, depletion of CD8^+^ T cells resulted in a partial loss of protection against rechallenge, including protection against reinfection by CD8^+^ T_RM_ cells in the URT **(** **Figure 4A** **)** (286). However, the role of antigen-specific CD4^+^ T memory cells in anti-SARS-CoV-2 function still deserves further study in animal models.

The study of the SARS-CoV-2 vaccine based on animal models can not only mitigate clinical risks but also provide conditions for the *in vivo* testing of vaccines and the design of immunization programs. Therefore, a variety of vaccines, including adenovirus vector vaccines (84, 85), DNA vaccines (84), mRNA vaccines (81), inactivated vaccines (31), and subunit vaccines (80), have been validated in animal models. However, whether vaccine-induced immune responses and immune memory are more effective in controlling infection than natural immunity remains a matter of concern (287). Current studies have demonstrated that both vaccination and infection produce up to 95% protection (288–292); however, peak neutralizing antibody responses produced by the vaccine ranged from approximately 1/2 to four times those in convalescent patients (153, 233, 293). Antibody decay rates did not differ significantly between vaccinated and naturally infected patients, with half-lives around 60 days (153). Repeated boosting with the vaccine may be beneficial to generate a longer period of protection than natural immunity. Although SARS-CoV-2 can evade immunity and reduce vaccine-induced protection by altering the epitopes recognized by neutralizing antibodies (190–193), omicron strains have evolved to exert low pathogenicity and high transmissibility (4–6, 294). This creates a complex situation, and it remains to be seen whether a new and more pathogenic SARS-CoV-2 will emerge in the future. Therefore, expanding protection against different variant strains may be an important requirement for next-generation vaccines.

## Conclusions and future perspectives

The global COVID-19 pandemic has caused a global recession and tremendous casualties. Although preventive vaccines for COVID-19 are being developed at an unprecedented rate, we still face many challenges owing to the large genome and high levels of genetic variation of SARS-CoV-2. Currently, there is no evidence that the innate immune response can control primary SARS-CoV-2 infection. Conversely, the loss of control of the innate immune response caused by SARS-CoV-2 immune escape and immune hijacking may correlate with severe clinical disease (9–11). Adaptive immune responses have demonstrated unique advantages in controlling SARS-CoV-2 infection and clinical disease severity (140–142). However, there are still many mysteries regarding the role of adaptive immunity in controlling SARS-CoV-2 infection. The rapid development of SARS-CoV-2 animal models has provided us with a good platform to simulate human clinical studies to understand the mechanism of virus infection and host immune effects, which play an irreplaceable key role in testing the effectiveness of vaccines and drugs.

At present, a variety of animal models ranging from rodents to NHPs have shown clinical symptoms similar to those of human COVID-19 when infected with SARS-CoV-2. However, there is still a gap between animal models and humans in terms of clinical characteristics, and it is difficult to comprehensively summarize the clinical symptoms of COVID-19 patients. However, the application of animal models provides a wealth of insights into SARS-CoV-2 pathogenesis, especially as the NHP model can faithfully reproduce COVID-19 pathology in humans. Although much research has been devoted to the adaptive immune response to SARS-CoV-2, there are still many unanswered questions that need to be further explored using animal models.

- What is the mechanism of lymphopenia caused by SARS-CoV-2 infection? Are there certain viral proteins that antagonize adaptive immune responses?
- Are neutralizing antibodies, CD4^+^ T cells, or CD8^+^ T cells necessary for the control of primary viral infection?
- What are the immune protection thresholds and persistence of humoral and cellular immune responses induced by vaccines and infections?
- How does aging affect resistance to SARS-CoV-2 through adaptive immunity?
- Is preexisting cross-immunity sufficiently effective against infection and high pathogenicity caused by SARS-CoV-2?
- What is the antiviral mechanism at play in asymptomatic patients? Is it related to preexisting cross-immunity?
- What is the role of tissue, especially lung-resident, immune responses in anti-SARS-CoV-2 activity and their relationship to circulating immune responses?

In conclusion, although the pathogenicity of omicron variants has been greatly reduced, the long-term existence and high mutation frequency of SARS-CoV-2 require constant attention to its threat to humans. Even though both SARS-CoV-2 and influenza virus can cause respiratory diseases, SARS-CoV-2 far exceeds influenza virus in many aspects, such as mutation rate, transmission characteristics, and induction of mortality. We need a better understanding of SARS-CoV-2 to better prevent the establishment of future novel variants of SARS-CoV-2.

## Author contributions

XW and JL conceived, wrote, and revised the study. NR drew the figures. All authors contributed to the article and approved the submitted version.

## Funding

This work was supported financially by grants from the National Natural Science Foundation of China (NSFC) (92169210 and 32200752).

## Conflict of interest

The authors declare that the research was conducted in the absence of any commercial or financial relationships that could be construed as a potential conflict of interest.

## Publisher’s note

All claims expressed in this article are solely those of the authors and do not necessarily represent those of their affiliated organizations, or those of the publisher, the editors and the reviewers. Any product that may be evaluated in this article, or claim that may be made by its manufacturer, is not guaranteed or endorsed by the publisher.

## Abbreviations

SARS-CoV-2, severe acute respiratory syndrome coronavirus 2; COVID-19, coronavirus disease 2019; SARS-CoV, severe acute respiratory syndrome coronavirus; MERS-CoV, Middle East respiratory syndrome coronavirus; NHP, nonhuman primate; ACE2, angiotensin-converting enzyme 2; hACE2, human ACE2; URT, upper respiratory tract; TMPRSS2, transmembrane serine protease 2; cTfh, circulating T follicular helper cells; CTL, cytotoxic T lymphocyte; B_RM_, tissue-resident memory B; T_RM_, tissue-resident memory T; RBD, receptor-binding domain.
